# Effects of Dietary Protein on Body Composition in Exercising Individuals

**DOI:** 10.3390/nu12061890

**Published:** 2020-06-25

**Authors:** Jose Antonio, Darren G. Candow, Scott C. Forbes, Michael J. Ormsbee, Patrick G. Saracino, Justin Roberts

**Affiliations:** 1 Department of Health and Human Performance, Nova Southeastern University, Davie, FL 33314, USA; 2 Faculty of Kinesiology and Health Studies, University of Regina, Regina, SK S4S0A2, Canada; Darren.candow@uregina.ca; 3 Faculty of Education, Department of Physical Education, Brandon University, Brandon, MB R7A6A9, Canada; forbess@brandonu.ca; 4 Department of Nutrition, Food & Exercise Sciences, Institute of Sports Sciences & Medicine, Florida State University, Tallahassee, FL 32313, USA; mormsbee@fsu.edu (M.J.O.); pgs16@my.fsu.edu (P.G.S.); 5 Discipline of Biokinetics, Exercise and Leisure Sciences, University of KwaZulu-Natal, Durban 4041, South Africa; 6 Cambridge Centre for Sport and Exercise Sciences, Anglia Ruskin University, Cambridge CB11PT, UK; Justin.roberts@anglia.ac.uk

**Keywords:** exercise, diet, amino acids, training

## Abstract

Protein is an important component of a healthy diet and appears to be integral to enhancing training adaptations in exercising individuals. The purpose of this narrative review is to provide an evidence-based assessment of the current literature examining increases in dietary protein intake above the recommended dietary allowance (RDA: 0.8 g/kg/d) in conjunction with chronic exercise on body composition (i.e., muscle, fat and bone). We also highlight acute and chronic pre-sleep protein studies as well as the influence of exercise timing on body composition. Overall, a high-protein diet appears to increase muscle accretion and fat loss and may have beneficial effects on bone when combined with exercise. Pre-sleep protein is a viable strategy to help achieve total daily protein goals. Importantly, there appears to be no deleterious effects from a high-protein diet on muscle, fat or bone in exercising individuals.

## 1. Introduction

Protein is an important component of a healthy diet and is essential for growth and development. Inadequate dietary protein, even with adequate caloric intake, will result in malnutrition [1]. Beyond health, dietary protein intake appears to be integral for exercising individuals [2,3,4,5,6,7]. Despite scientific inquiry by von Liebig in 1842 [8], it has only become increasingly evident in recent decades that protein requirements are elevated above the recommended dietary allowance (RDA) of 0.8 g/kg/d [9]. These greater dietary protein needs may be associated with exercise-induced mitochondrial biogenesis, protein synthesis, and endocrine and immune responses [5,10]. The purpose of this brief narrative review is to examine the efficacy and safety of increasing dietary protein intake in trained individuals, as well as the acute and chronic effects of pre-sleep protein on body composition. Lastly, we evaluate the current scientific literature pertaining to increasing protein intake on bone.

## 2. Dietary Protein and Body Composition in Trained Individuals

For individuals who exercise regularly, the current body of evidence supports dietary protein needs 1.5- to 2.0-fold higher than the RDA of 0.8 g/kg/d [4,5,6,10,11]. It is important to note that these elevated dietary protein requirements and recommendations are for individuals performing chronic exercise (>3 months) [9]. Gontzea et al. was the first to demonstrate an increased protein requirement, based on a negative shift in whole-body nitrogen balance, in participants initiating an exercise training program and ingesting a constant protein intake of 1.0 g/kg/d [12]. As such, the primary emphasis of this section will be on studies examining dietary protein needs for chronic exercising individuals. Thus, sedentary, unhealthy, or inactive populations performing short-term training (<3 months) will not be discussed; for clinical trials discussing sedentary populations, see the following references [13,14,15,16].

High-protein diets may assist with fat mass reduction through a variety of mechanisms including enhanced resting and sleeping energy expenditure [17], elevated activity related energy expenditure, increases in non-exercise activity thermogenesis [18,19] and a greater thermic effect of feeding relative to other macronutrients [20,21]. Furthermore, it is well known that protein feeding acutely elevates muscle protein synthesis post-exercise [22,23,24,25,26,27,28,29], which in theory should result in greater muscle protein accretion over time. However, caution is required when extrapolating these ‘snap-shot’ findings to whole-body adaptations [30]. In addition, it is important to note that these acute feeding studies are rigorously controlled (i.e., performed in a fasted state, single-protein-only dose) to enhance internal validity; however, the generalizability (i.e., external validity) of these studies in free-living humans consuming mixed meals multiple times a day may be limited. As such, longitudinal controlled trials may be the ideal methodology to determine whether protein doses above the RDA can impact body composition.

Kerksick et al. [31] assessed the effects of a 10 week (4 d/wk) resistance training program in conjunction with whey protein supplementation on body composition, muscular strength, muscular endurance, and anaerobic capacity. Resistance-trained males (*N* = 36) received either 48 g/d of carbohydrate (placebo), or 40 g/d of whey protein + 8 g/d of casein (WC), or 40 g/d of whey protein + 3 g/d of branched-chain amino acids + 5 g/d of L-glutamine (WBG). There were no differences between groups for energy intake, training volume, blood parameters, anaerobic capacity, or muscular strength. WC experienced the greatest increases in lean body mass (as assessed by dual energy x-ray absorptiometry; DXA), possibly due to the higher complete protein (whey and casein) intake [31]. Ormsbee et al. examined protein supplementation on muscular strength and body composition during a 6 month (5 d/wk) concurrent strength and endurance training program in sedentary men and women [32]. Participants (*N* = 51; 26 males, 25 females; age 18–25 yr) were randomized to a protein (PRO; 42 g/serving) or carbohydrate (CON) supplement twice daily. Females in both groups similarly improved lean body mass, one-repetition maximum (1-RM) bench press and hip sled strength. Importantly, females supplementing with PRO experienced a greater reduction in fat mass than CON (PRO: −1.7 ± 0.5 kg vs. CON: 0.1 ± 0.5 kg). Males experienced similar changes in body composition [32].

In a series of studies, Antonio et al. examined the impact of high-protein diets with and without alterations in habitual training [33,34]. The first study included trained (*N* = 30) males and females consuming 4.4 g/kg/d of protein for eight weeks and reported no changes in body mass, muscle or fat mass. Participants were instructed not to change their habitual training throughout the study. Thus, the follow-up study assessed a high-protein diet (3.4 g/kg/d) in conjunction with a periodized heavy resistance-training program [34]. Participants (*N* = 48) in the control and high-protein groups were instructed to consume ~2 and >3 g/kg/d of dietary protein, respectively. Despite the high-protein group consuming more calories (~495 kcals/d), they experienced a greater decrease in fat mass (−1.6 kg) compared to control (−0.3 kg). On the other hand, the high-protein (+1.5 kg) and control groups (+1.5 kg) gained a similar amount of lean body mass. Furthermore, no deleterious effects were reported in either group [34]. Similarly, Campbell et al. randomized aspiring female physique competitors (*N* = 17) to a high-protein (2.5 g/kg/d) and low-protein (0.9 g/kg/d, similar to the RDA: 0.8 g/kg/d) diet in conjunction with 8 weeks of resistance training [35]. Following training, fat-free mass increased more in the high-protein group (2.1 kg) compared with the low-protein group (0.9 kg). In addition, fat mass decreased more in the high-protein group (−1.1 kg) compared to the low-protein group (−0.7 kg). There were no differences in strength changes between groups [35]. Thus, protein intakes greater than twice the RDA may improve body composition in trained individuals that alter their training regimen. Furthermore, the addition of protein (31 g/d of milk protein) in physically active older adults (67–73 yr) with relatively low habitual dietary protein consumption (<1 g/kg/d) increased lean body mass and reduced fat mass over a 12 week period compared to those who did not augment their diet with additional protein [36]. In addition, whey protein supplementation (77 g/d) in military participants increased upper-body muscle endurance and decreased fat mass over 8 weeks

In contrast, there have been several studies showing no benefit from a higher dietary protein intake. In novice bodybuilders (*N* = 12; 22 yr) engaged in their initial stages of training, a high protein intake of 2.62 g/kg/d compared to 1.35 g/kg/d did not alter muscle mass or strength gains over 3.5 weeks [37]. Antonio et al. also found no effect from either 2.6 to 3.3 g/kg/d of protein during resistance training on body composition in resistance-trained young males [38,39]. Spillane and Willoughby randomized (*N* = 21) resistance-trained males to either a protein (94 g/d) plus carbohydrate (196 g/d) or carbohydrate alone for 56 days and found no differences between groups over time on body composition [40]. In a cross-sectional study, collegiate strength/power athletes (*N* = 23) performed 12 weeks of resistance training in which they were stratified into three groups based on habitual daily protein intake below recommended levels (BL; 1.0–1.4 g/kg/d; *n* = 8), recommended levels (RL; 1.6–1.8 g/kg/d; *n* = 7) and above recommended levels (AL; > 2.0 g/kg/d; *n* = 8). There were no changes in body mass, lean body mass or fat mass in any group and similar improvements in 1-RM bench press and 1-RM squats between groups [41]. Collectively, there appears to be a small but beneficial effect of a high protein intake (≥2x the RDA) on lean body mass and in trained individuals. This is in agreement with a recent systematic review, meta-analysis, and meta-regression [42]. Morton et al. assessed data from 49 studies with 1863 participants and found that protein supplementation significantly increased fat-free mass as well as muscle fiber cross-sectional area [42]. Conversely, there is limited data to suggest that protein supplementation may promote a loss of fat mass. Thus, the pragmatic approach regarding dietary protein recommendations should err on the higher side of intake (i.e., at least twice as much as the RDA and perhaps even higher). Further, importantly, increasing dietary protein intake had no deleterious effects. Future research should clarify what the upper limit of protein intake might be regarding gains in lean body mass; conversely, it is possible that the amount of protein that is required for lean body mass accretion may not be the same as what is needed for the loss of fat mass.

## 3. Pre-sleep Protein

In trained individuals, consuming low-energy protein-centric food items prior to sleep provides an opportunity to increase energy intake, support the muscle repair process, and provide an additional time to achieve total daily protein goals [43,44,45]. The purpose of this section is to summarize the acute and chronic effects of pre-sleep protein as well as discuss the impact of exercise timing in relation to pre-sleep protein on body composition changes.

### 3.1. Acute Dosing

Multiple studies indicate that pre-sleep protein “meals” have either a beneficial or no significant impact on overnight metabolism without altering fat oxidation [46,47,48,49,50,51]. Groen et al. was the first to show, in a proof-of-concept study, that pre-sleep dietary protein is well digested and absorbed, resulting in an increased overnight net protein balance in elderly adults [52]. Follow-up studies in younger populations provided further support [53]. The hyper-aminoacidemia from pre-sleep protein resulted in an increase in overnight muscle protein synthesis [52,54]. When previous evening exercise is performed, greater amounts of amino acids from pre-sleep dietary protein are incorporated into myofibrillar protein [55], resulting in an augmented muscle protein synthetic response [53]. While no optimal dose has been determined for pre-sleep protein consumption, 30 g of casein, with or without 2 g of free leucine, increased net muscle protein balance without influencing post-exercise fractional synthetic rate (FSR) overnight [56]. However, Res et al. showed an increase of ~22% in mixed muscle FSR when 40 g of pre-sleep casein protein was consumed compared to placebo following an acute bout of lower-body resistance training in young, recreationally active males [53]. Furthermore, no alterations in sleep quality with pre-sleep protein ingestion were reported [56,57,58]. Using microdialysis, no alterations in overnight fat metabolism with pre-sleep protein ingestion in males [59] or trained females [58] were found. Overall, acute studies support that pre-sleep protein represents an additional opportunity to stimulate the synthetic response without a detriment to fat metabolism.

### 3.2. Long Term

While most studies have shown benefits with sufficient doses of pre-sleep protein alone and in conjunction with evening exercise, fewer studies exist for chronic pre-sleep protein consumption with mixed results. van Loon’s group were the first to show that a pre-sleep protein (27.5 g casein) and carbohydrate (15 g) beverage led to greater muscle strength gains and ~10% increase in quadriceps cross-sectional area compared to a ~6% increase in the non-caloric placebo (*p* < 0.05) in recreationally active young men after 12 weeks of whole-body resistance training performed in the evening [60]. In contrast, Vangsoe et al. reported no improvements for strength or body composition in active young males when 0.4 g/kg insect protein (~32 g on average) was ingested pre-sleep compared to iso-energetic CHO following 8 weeks of whole-body resistance training [61]. Interestingly, both studies reported relative protein intakes (means between 1.3–1.6 g/kg/d prior to supplementation) well above the RDA and within previously suggested optimal ranges [62] despite not providing standardized nutrition during the training intervention. Snijders et al. performed resistance training in the evening hours [60]. Though exercise time was not controlled in the Vangsoe et al. study, the authors indicated most performed their exercise sessions in the afternoon. This methodological difference in exercise time, and therefore proximity of pre-sleep protein ingestion to the exercise bout may explain discrepancy in findings. However, in both of these studies, participants were not in nitrogen balance and, consequently, the protein-supplemented groups consumed greater relative protein intakes compared to the control groups. In order to determine whether pre-sleep protein is advantageous or simply consuming greater daily protein is key, Antonio et al. reported no difference between morning (prior to 1200 h) or night-time (≤90 min pre-sleep) protein intake (54 g casein) on body composition or strength outcomes following 8 weeks of strength training in trained males and females [63]. In a similar study design, Joy et al. reported no difference between 10 weeks of morning and pre-sleep casein on muscle size or strength in recreationally active males [64]. In contrast, casein consumed in the morning (1000 h) and evening (2230 h) improved fat-free mass to a greater extent than morning (1000 h) and afternoon (~1550 h) in high school-aged boys performing resistance exercise [65]. At this time, it is not entirely clear how significant timing is regarding changes in body composition.

### 3.3. Exercise Timing

Recently, investigators have shown benefits of pre-sleep protein on muscle recovery following evening exercise [66,67]. Using a cross-over design, West et al. provided 25 g of a whey protein blend or energy matched carbohydrate (CHO) to young, resistance-trained males following whole-body resistance training. During the 24 h recovery period, net protein balance was improved and there was a moderate beneficial effect on maximal voluntary contraction, repetitions to failure, and Wingate peak power [66]. Similarly, Abbott et al. utilized a cross-over design and provided 40 g of pre-sleep casein or CHO to professional soccer players following night-time soccer matches. There was no clear difference in external loads between matches. In the 12 h recovery period, casein most likely improved reactive strength index, muscle soreness, and counter movement jump (CMJ) with likely beneficial effects at 36 h post-exercise for CMJ and most likely beneficial effects at 36 h for reactive strength index performance [67]. Others have challenged the efficacy of pre-sleep protein [63,64,68,69,70] on muscular adaptations and performance. When exercise is performed in the morning, no benefits are reported for pre-sleep protein [68,69,70]. Of the three studies utilizing morning exercise and pre-sleep protein, two used trained populations for their investigation. Apweiler et al. had young, active males and females perform 100 drop jumps in the morning (0730–0930 h) to induce muscle damage, then consume either 40 g pre-sleep casein or CHO 30 min before bed. As expected, the muscle damaging protocol induced muscle soreness and reduced pain pressure threshold, maximal isometric voluntary contraction (MIVC), and CMJ, indicating muscle damage. However, there were no differences between groups or sexes for any measure [70]. Similarly, in order to mimic an endurance training camp, Larsen et al. had 32 trained runners perform 11 exercise bouts during a 1 week period while consuming 0.5 g/kg (~37 g) of either whey protein isolate or CHO each night before bed. Standardized nutrition was provided by the researchers with protein for both groups set at 1.8 g/kg. Thus, protein supplementation was provided hours after the training stimulus. Again, no benefit was observed for time trial performance, training volume, or blood biomarkers between groups [68].

While pre-sleep protein seems to improve recovery from late-evening exercise, there does not seem to be a benefit on next-day exercise performance [47,49]. Ormsbee et al. provided 355 mL of fat-free chocolate milk (12 g PRO, 30 g CHO) or non-nutritive placebo 30 min pre-sleep to trained female runners and triathletes in a double-blind, cross-over design. This study observed most likely trivial improvements in next-day 10 km time trial performance between treatments [47]. It is important to note that they used a low-calorie and low-protein option for pre-sleep feeding, which likely contributed to the null findings. Similarly, in a double-blind cross-over study, Madzima et al. [49] provided non-nutritive placebo, 24 g, and 48 g of pre-sleep casein and whey to active, young women for a total of five treatment conditions. These authors reported no clear effects of any type or dose of pre-sleep protein compared to placebo on next-morning resistance exercise volume. However, the effects of pre-sleep protein ingestion on next-day performance in men is presently unknown. The work to date on pre-sleep protein feeding has all utilized animal-based protein. Given the rise in popularity of plant-based proteins, more research is required to investigate the effects of these proteins consumed pre-sleep. It has been suggested that combining plant-based proteins may overcome any essential amino acid deficiencies and therefore provide a complete amino acid profile [71] similar to animal-based protein [72]. Future work should compare pre-sleep animal-based protein with combinations of plant-based protein sources.

## 4. Protein and Bone in Young, Exercising Individuals

It is well established that an increase in habitual dietary protein intake (including supplementation) above the dietary RDA (0.8 g/kg/d) has a beneficial effect on muscle mass and muscle performance [42]. However, the potential beneficial effects of increased protein intake on properties of bone are less clear.

Proteins comprise approximately 30–50% of bone mass and volume [73] and may play a crucial role in bone accretion and bone strength over time [74]. In summarizing several meta-analyses and review articles, a report endorsed by the European Society for Clinical and Economical Aspects of Osteoporosis, Osteoarthritis, and Musculoskeletal Diseases concluded that increasing dietary protein intake was associated with an increase in bone mineral density and a reduction in hip fracture risk [75]. While the mechanisms explaining the potential beneficial effects of protein on bone remain to be determined, protein influences calcium kinetics, growth factors and hormones, and muscle mass [76]. Increasing protein consumption typically increases calcium excretion due to improved intestinal calcium absorption but has no negative effect on calcium homeostasis [77]. An increase in calcium absorption would theoretically decrease parathyroid hormone secretion which would subsequently increase blood calcium levels and have a favorable effect on net bone accrual by suppressing bone resorption. Furthermore, increasing amino acid uptake increases collagen synthesis and osteocalcin expression, leading to a positive effect on osteoblast differentiation (cells involved in bone formation) [78]. In addition to calcium kinetics, protein augments insulin-like growth factor 1, a growth factor highly involved in bone growth [77,78], and increases the release of incretin hormones which have anabolic effects on bone mass and microarchitecture (bone strength) [79]. Furthermore, it is well established that dietary protein ingestion increases the rates of muscle protein synthesis, which could lead to greater muscle accretion over time [42]. Greater muscle mass may increase mechanical strain on bone at their tendon attachment sites, leading to net bone accretion over time [80].

The vast majority of research investigating the effects of dietary protein consumption on properties of bone have focused on aging adults and disease state populations. Very limited research exists regarding younger exercising individuals. This focus area is important because exercising individuals are at a greater risk of developing/acquiring stress fractures [81] possibly due to an imbalance between bone resorption and bone formation caused by prolonged or high-intensity exercise [82], which over time may negatively influence training frequency, duration and progression. Therefore, strategies which increase bone mass and strength and subsequently decrease the prevalence of stress fractures are important for exercising individuals.

Research is mixed regarding the efficacy of increased dietary protein consumption (including supplementation) on properties of bone in exercising individuals. Male endurance runners (28 ± 6 yr; ≥2 years of experience) who consumed a protein-containing beverage (0.5 g/kg/d) immediately after exercise (treadmill run time to exhaustion at 75% VO_2_ max) experienced a reduction in C-terminal telopeptides of type I collagen by 22–61% (indicator of bone resorption) and an increase in N-terminal propeptides of type I procollagen by 1–3% (indicator of bone formation), thereby creating a net positive bone turnover balance [82]. Similarly, in young swimmers (11–17 yr; ≥1 year of training and competition experience), post-exercise whey protein ingestion (2 × 0.3 g/kg/d) significantly decreased an indicator of bone resorption by 22% (carboxy-terminal collage cross-links) compared to a carbohydrate-based placebo. Protein ingestion had no effect on procollagen type 1 intact N-terminal propeptide levels (indicator of bone formation) [83]. In examining the effects of low (14%) and high (44%) whey protein supplementation (consumed immediately before and after whole-body resistance training sessions) for 12 weeks (3x/week) in collegiate male baseball players (~20 years of age; >10 years of training experience), Chung et al. observed a significant increase in humerus bone mineral density in the dominant limb from high protein consumption only (high-protein: pre 1.34 ± 0.03 g/cm^2^, post 1.37 ± 0.02 g/cm^2^, 2.2%; low-protein: pre 1.32 ± 0.03 g/cm^2^, post 1.33 ± 0.03 g/cm^2^, 0.8%; *p* < 0.05) [84]. However, there was no effect from protein, independent of dosage, on whole-body bone mineral density. Furthermore, there was no evidence of a deleterious effect from protein on bone. Higher intake of dairy-based protein food products (i.e., milk) was associated with a reduced rate of stress fractures in trained young female cross-country athletes who ran approximately 40 miles per week [85]. Milk ingestion was also a significant predictor of hip bone mineral density and whole-body bone mineral content accrual. It is important to note that dairy-based protein food products contain, in addition to protein, calcium, vitamin D, carbohydrates and fats. Interestingly, animal protein intake predicted gains in whole-body bone mineral density and content. The authors speculate that the beneficial effects from dairy-based protein food products on bone density may be reflective of the animal protein content (i.e., 47% of animal protein per day came from dairy consumption) [86]. In examining the effects of high protein consumption (habitual dietary consumption and/or supplementation; 2.8 g/kg/d or ~169 ± 55 g/d) vs. habitual dietary protein consumption (1.5 g/kg/d or ~91 ± 17 g/d) in young females (~36 yr) who had been performing resistance and/or aerobic exercise 3x/week for ≥1 year, Antonio et al. found no effect (anabolic or deleterious) from additional dietary protein on whole-body bone mineral density (high protein: pre 1.25 ± 0.11 g/cm^2^, post 1.24 ± 0.101 g/cm^2^; habitual protein: pre 1.22 ± 0.08 g/cm^2^, post 1.22 ± 0.09 g/cm^2^) or content (high protein: pre 2.55 ± 0.38 kg, post 2.53 ± 0.40 kg; habitual protein: pre 2.47 ± 0.35 kg, post 2.47 ± 0.34 kg) after 6 months [87]. After 12 months, high protein consumption (2.3 g·kg^−1^·d^−1^) still had no effect (anabolic or deleterious) on whole-body bone mineral density (1.21 ± 0.09 g/cm^2^) or content (2.4 ± 0.3 kg). [88]. Finally, whey (40 g) and casein (8 g) protein supplementation during 10 weeks of high-intensity resistance training in trained young males (defined as performing > 3 h of resistance training per week for >1 year) had no effect on whole-body bone mineral content (protein: pre 2.45 2.4 ± 0.4 kg, post 2.47 ± 0.4 kg; placebo: pre 2.54 ± 0.5 kg, post 2.55 ± 0.6 kg) [31].

Collectively, limited research indicates that protein supplementation can positively influence bone turnover and regional bone mineral in some exercising individuals. Increasing habitual dietary protein intake above the recommended daily allowance of 0.8 g/kg/d has no deleterious effects on bone in exercising individuals. Future research should investigate the long-term effects of dietary protein, with and without exercise training, on bone mineral, strength and geometric measures in exercising individuals.

## 5. Conclusions

It is evident that total protein intake is an important factor for any alteration in body composition. Based on the current evidence, protein intakes that far exceed the RDA may promote additional gains in lean body mass as well as a decrement in fat mass. However, in order to best achieve a gain in lean body mass or loss of fat mass, this is best achieved when complimented with a rigorous resistance training program. The role of pre-sleep protein feedings is not entirely understood. Nonetheless, pre-sleep feedings may be beneficial to athletes inasmuch as it contributes to total daily protein intake. To date, higher protein intakes tend to have a neutral effect on bone mineral density; however, there is evidence to suggest that it can indeed have a positive effect.
